# Supplementary material for: Outcomes of High-Grade Cervical Dysplasia with Positive Margins and HPV Persistence after Cervical Conization
Source: Vaccines (Basel). 2023 Mar 18;11(3):698. doi: 10.3390/vaccines11030698 (PMC10051663; doi:10.3390/vaccines11030698)
Supplement: Supplementary file 1 [file vaccines-11-00698-s001.zip › vaccines-2162953-supplementary.pdf]

Table S1. Severity of the lesion: CIN2 vs. CIN3.

	**CIN 2 (n=61)**	**CIN 3 (n=102)**	**p value**
Age	42.4 (26-61)	43 (74-20)	0.763
BMI	22.8 (19-33)	26.1 (42-18,4)	<0.001
HPV type			
HPV 16/18	21 (34.4%)	52 (50.9 %)	0.039
Others HR-HPV	4 (6.5%)	25 (24.5 %)	0.003
LR-HPV & Unknown	36 (59%)	25 (24.5 %)	<0.001
LEEP	60 (98.3%)	101 (99 %)	0.711
LASER	1 (0.7%)	1 (1%)	0.711
Ectocervical positive margins	54 (88.5%)	65 (63.7%)	<0.001
Endocervical positive margins	7 (11.5%)	37 (36.3%)	<0.001
HPV persistence	61 (100%)	102 (100%)	1.00
HPV vaccination after treatment	10 (16.3%)	15 (14.7%)	0.772
Recurrence	2 (3.2%)	15 (14.7%)	0.020

Table S2. Positive surgical margins: ectocervical vs. endocervical.

	**Positive ectocervical margins (n=119)**	**Positive endocervical margins (n=44)**	**p value**
Age	42.68 (24-74)	43.43 (20-63)	0.7117
BMI	23.61 (18.4-42)	27.22 (19-42)	<0.001
HPV type			
HPV 16/18	44 (36.9%)	29 (65.9%)	0.001
Others HR-HPV	19 (15.9%)	10 (22.7%)	0.316
LR-HPV & Unknown	56 (47%)	5 (11.3%)	<0.001
LEEP	118 (99.1%)	44 (100%)	0.5419
LASER	1 (0.9%)	0 (0 %)	0.5419
CIN 2	54 (45.3%)	7 (15.9%)	<0.001
CIN 3	65 (54.6%)	37 (84%)	<0.001
HPV persistence	119 (100%)	44 (100%)	1.00
HPV vaccination after treatment	9 (7.5%)	6 (13.6%)	0.235
Recurrence	6 (5%)	12 (27.2%)	<0.001

Table S3. HPV16/18 vs. other high-risk HPV positivity.

	**HPV16/18 positivity (n=73)**	**Other high-risk HPV positivity (n=28)**	**p value**
Age	41.86 (20-62)	42.57 (25-63)	0.797
BMI	24.61 (18.4-35)	23.28 (21-26)	0.156
CIN 2	21 (28.7 %)	3 (10.7%)	0.056
CIN 3	52 (71.2%)	25 (89.2%)	0.056
LEEP	73 (100%)	28 (100%)	
LASER	0 (0 %)	0 (0%)	0.999
Exocervical positive margins	44 (60.2%)	18 (64.2%)	0.710
Endocervical positive margins	29 (39.7%)	10 (35.7%)	0.710
HPV persistence	73 (100%)	28 (100%)	1.00
Persistence after 6 months	73 (100%)	28 (100%)	1.00
Recurrence	8 (10.9%)	3 (10.7%)	0.971
